# Arterial stiffness assessment using PPG feature extraction and significance testing in an in vitro cardiovascular system

**DOI:** 10.1038/s41598-024-51395-y

**Published:** 2024-01-23

**Authors:** Redjan Ferizoli, Parmis Karimpour, James M. May, Panicos A. Kyriacou

**Affiliations:** Research Centre for Biomedical Engineering, City, University of London, London, EC1V 0HB UK

**Keywords:** Biomedical engineering, Vascular diseases, Arterial stiffening

## Abstract

Cardiovascular diseases (CVDs) remain the leading cause of global mortality, therefore understanding arterial stiffness is essential to developing innovative technologies to detect, monitor and treat them. The ubiquitous spread of photoplethysmography (PPG), a completely non-invasive blood-volume sensing technology suitable for all ages, highlights immense potential for arterial stiffness assessment in the wider healthcare setting outside specialist clinics, for example during routine visits to a General Practitioner or even at home with the use of mobile and wearable health devices. This study employs a custom-manufactured in vitro cardiovascular system with vessels of varying stiffness to test the hypothesis that PPG signals may be used to detect and assess the level of arterial stiffness under controlled conditions. Analysis of various morphological features demonstrated significant (p < 0.05) correlations with vessel stiffness. Particularly, area related features were closely linked to stiffness in red PPG signals, while for infrared PPG signals the most correlated features were related to pulse-width. This study demonstrates the utility of custom vessels and in vitro investigations to work towards non-invasive cardiovascular assessment using PPG, a valuable tool with applications in clinical healthcare, wearable health devices and beyond.

## Introduction

Cardiovascular diseases (CVD) stand as the leading contributor to global mortality^1–3^. Within the spectrum of CVD are diseases impacting both the heart and blood vessels. Examples include coronary artery disease (CAD), heart attack, stroke, and even the ageing of the vascular system, which profoundly influences heart and vessel health. Considering the inevitable nature of vascular ageing, it becomes crucial to delve into this domain and introduce innovative approaches for evaluating the ageing process of blood vessels.

The term "vascular ageing" describes the alterations that take place in the blood vessels with age, such as a loss of elasticity, which can impair the vascular system's ability to operate efficiently. Arterial stiffness results from the arteries loss of elasticity, and this is an important indicator of vascular ageing^4^. Understanding the connection between vascular ageing and arterial stiffness will help better understand age-related disorders.

Photoplethysmography (PPG), an established optical sensing method, has enormous promise for assessing cardiovascular health^5–8^. In the well-established technique of pulse oximetry and in the majority of wearable devices, PPG is frequently employed to estimate blood oxygen saturation and heart rate^9^. Research has long established an association between changes in cardiovascular health and the PPG signal^10^. PPG is a technique which assesses volumetric changes; changes in vessel structure associated with arterial stiffness can reduce vessel expansion, leading to potential disruptions in blood flow volume. This implies that PPG has the capability to identify signs of arterial stiffness and can be utilised as a primary tool to study the vascular system^11^.

Photoplethysmography uses light to monitor volumetric changes in blood vessels non-invasively^12^. The light source(s), which are commonly light emitting diodes (LEDs), and photodetectors (photodiodes or phototransistors) are the two primary parts of a PPG sensor. The photodetector measures the amount of light received^7^. The sensor can work in transmission mode or reflectance mode depending on where it is placed on the body. In transmission mode, red and infrared light from the LEDs travels through the body component and is picked up by the photodetector on the opposing side^13^. In reflectance mode, the photodetector measures the amount of light reflected^14^.

The simplicity of PPG and its inexpensive cost have led to its widespread adoption. With single-site PPG devices, continuous measurements are achievable since only one contact point is needed to take readings^15^. Another option is multi-site PPG, recorded simultaneously at several locations, such as the ears, fingers and toes, for the measurement of pulse transit time^16,17^. Previous studies have shown that multi-PPG and pulse transit time can be used to evaluate CAD, peripheral arterial disease (PAD), and ageing^18–20^. Pulse transit time measurements can also be measured by pairing PPG with other physiological signals, such as electrocardiography^21^. This experiment utilises single-site PPG for arterial stiffness assessment due to the practicality of having only one device and measurement point, for example a smartwatch recording at the wrist, which could be employed in clinical as well as consumer settings.

Ideal PPG signals feature a systolic and diastolic peak, separated by a dicrotic notch. However, in practice, the diastolic peak is often absent, as seen in older subjects^22,23^. This study employs feature extraction algorithms relying on the systolic peak alone from the PPG, relating the resultant features to arterial stiffness. Furthermore, the features analysed are based on the original filtered PPG wave, rather than the derivatives. As such, this correlation would be applicable in instances when the second derivative fiducial points are unavailable, which are used in existing pulse wave analysis methods^24^.

This paper aims to explore the relationship between arterial stiffness and vascular ageing by assessing PPG signals acquired from an in vitro vascular system, providing an indication of the utility of various pulse wave features, which would be beneficial to future arterial stiffness assessment techniques^25^ as well as exploring PPG-based arterial assessment in a controlled environment^26–28^, whereby arterial stiffness can be isolated as a factor and manipulated. The hypothesis being that certain morphological features will show significant indication of vessel stiffening.

## Methods

### Vessel manufacture

Manufacturing of the custom vessels involved two parts based on a method developed by Nomoni et al.^29^. Firstly, the elastomer (PlatSil Gel-10, Polytek Development Corp., Easton, PA) was formulated and mixed for the desired vessel geometry and mechanical properties; secondly the vessel was fabricated via a combined dip-coating and curing process, using commercial silicone tubing (Hilltop Products Limited, Warrington, UK) as the vessel form, Silicone was chosen due to its durability; in contrast to latex, silicone can maintain its form and endure high temperatures^30^, crucial for the heat curing process being used in this setup. Hardener (Polytek Development Corp., Easton, PA) was added in different amounts, from 5 to 25%, in steps of 5%, to the elastomer mixture to create five vessels of varying stiffnesses. The resultant vessels were tensile tested in a Universal Testing System (Instron 5944, Norwood, MA), indicating Young’s elastic modulus values of 0.52 MPa, 0.60 MPa, 0.64 MPa, 0.78 MPa and 0.80 MPa, with an inner diameter of 2.8 mm and a wall thickness of 0.5 mm. This was representative of the dimensions of the arteries found in the forearm^31–33^ and in the similar range of vascular elastic properties^34–37^.

### In vitro system, signal acquisition and analysis

**Figure 1:**
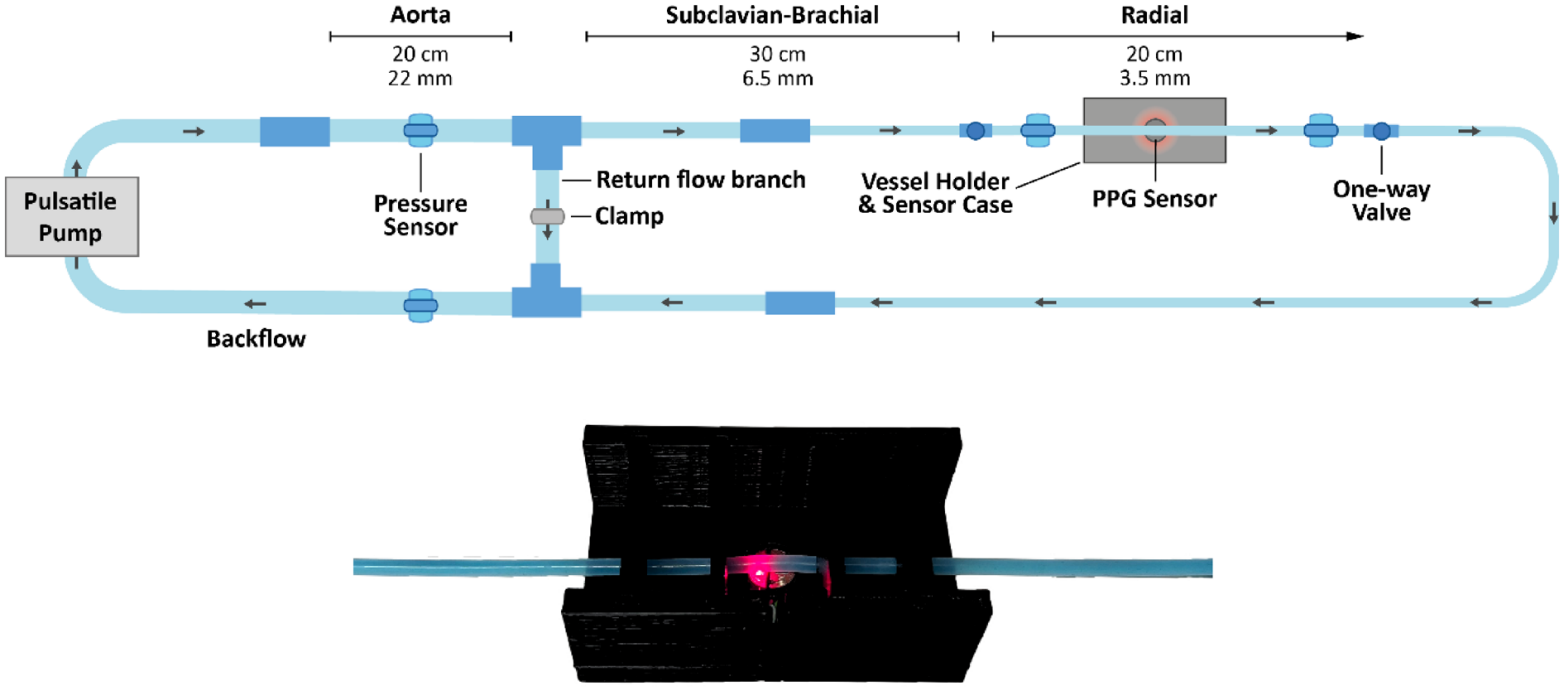
Schematic diagram of in-vitro setup (top) and photo of custom vessel held in PPG sensor case (bottom).

An in vitro setup was constructed using commercial silicone tubing that imitated the vessels found in the upper vascular system. Custom-manufactured portions were used in the areas being monitored. This setup was linked to a Pulsatile Pump System (PD-1100, BDC Laboratories, Wheat Ridge, CO). The pulsatile pump replicated the pulsatile flow characteristic of the human heart at 60 beats per minute. To mimic the optical properties of the blood and give good signals in the red and infrared wavelengths being utilised, a mixture of methylene blue powder (Thermo Fisher Scientific, UK) and deionised water was used, which was then circulated throughout the system. The custom vessels were integrated into this arrangement at locations approximating those in the vascular tree that were of interest, primarily the radial and ulnar arteries following the brachial artery bifurcation, and beneath them, a reflectance PPG sensor with a red (660 nm) and infrared (940 nm) LED was attached. The sensors were connected to a custom dual-channel PPG acquisition system with a sampling rate of 2000 Hz, created by our research group at the Research Centre for Biomedical Engineering (RCBE), City, University of London^18,38^. Raw signals were acquired by situating the sensors below the vessels, within a sensor casing to house the sensor and hold the vessel in place, shown in Fig. 1. Signals were displayed and recorded in LabVIEW (Version 2023 Q1, National Instruments, Austin, TX).

**Figure 2:**
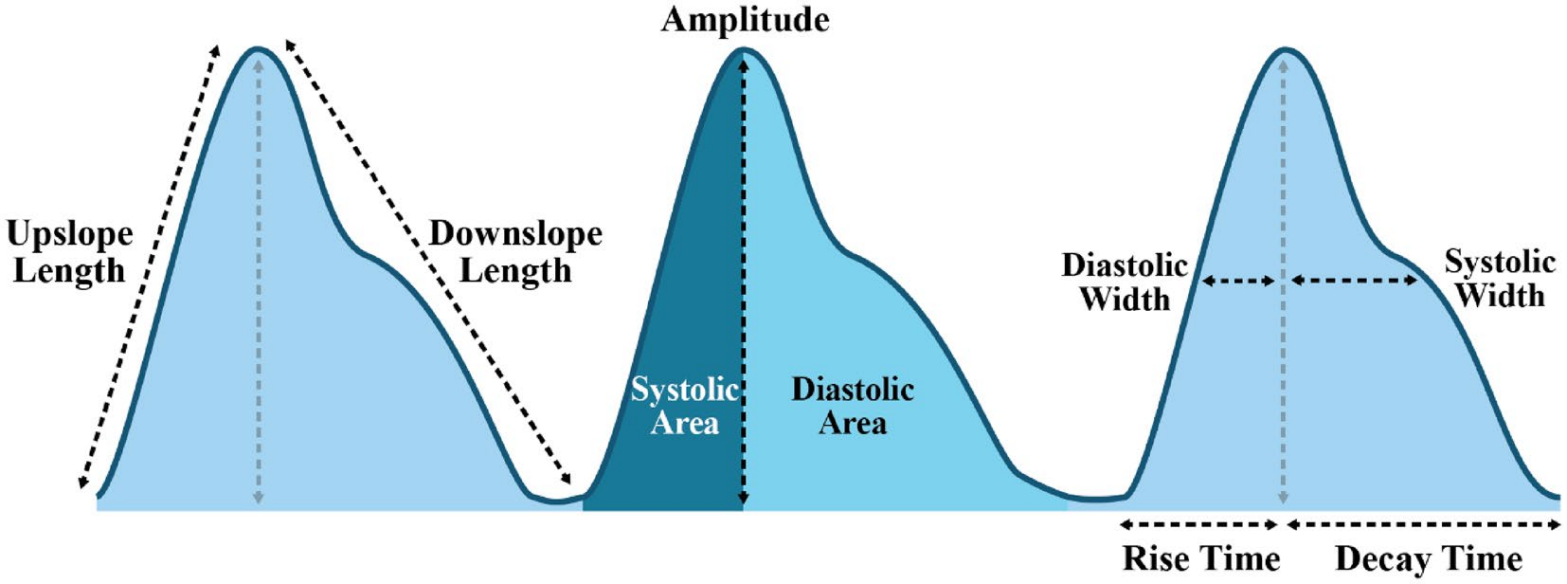
Example PPG features.

Feature extraction and analysis of the recorded signals, exemplified in Fig. 2, were performed using a custom Python script, also developed at the RCBE, University of London^39^. Recordings of 4 min from each vessel were split into 10 s windows, which were averaged to produce one data point per window. This produced a series of values for each feature, listed in Table 1. These results were illustrated with MATLAB (Version R2023a 9.14, MathWorks, Natick, MA) and statistically analysed and ranked by the Pearson correlation coefficient, through cross correlation computed in the R statistical programming language^40^.

**Table 1:** Table of PPG features.

Feature (abbreviation)	Description
Area Under Curve (AUC)	Area of pulse
Systolic Area Under Curve (S-AUC)	Area of the systolic phase of the pulse
Diastolic Area Under Curve (D-AUC)	Area of the diastolic phase of the pulse
Area Under Curve Ratio (AUC Ratio)	Ratio between systolic and diastolic areas
Rise Time	Time taken for signal to rise from trough to peak
Decay Time	Time taken for signal to fall from peak to trough
Rise-Decay Time Ratio	Ratio of decay time to rise time
Amplitude	Median amplitude
Upslope Length	Length of tangent from trough to peak
Downslope Length	Length of tangent from peak to trough
Upslope	Gradient of pulse on rising edge
Downslope	Gradient of pulse on falling edge
Onset-End Slope	Rate of change of the pulse over the entire pulse length
Slope Ratio	Rising slope gradient (upslope) compared to falling slope gradient (downslope)
Length-Height Ratio	Ratio of the height of a pulse compared to its width
Slope Length Ratio	Ratio of the upslope length of a pulse compared to its downslope length
Upslope Length Ratio	Proportion of the total pulse length that is made up of the rising phase
Downslope Length Ratio	Proportion of the total pulse length that is made up of the falling phase
Start Datum Area	Area between PPG signal and the tangent from trough to peak (systolic phase)
End Datum Area	Area between PPG signal and the tangent from peak to trough (diastolic phase)
Datum Area Ratio	Ratio between systolic and diastolic datum areas
Max Start Datum Difference	Maximum length between PPG signal and peak-trough tangent (systolic phase)
Max End Datum Difference	Maximum length between PPG signal and trough-peak tangent (diastolic phase)
Median Start Datum Difference	Median length between PPG signal and peak-trough tangent (systolic phase)
Median End Datum Difference	Median length between PPG signal and trough-peak tangent (diastolic phase)
Pulse Width	Width of the entire pulse of one cycle (measured at 50% of height)
Systolic Width	Width of the systolic phase of the pulse (measured at 50% of height)
Diastolic Width	Width of the diastolic phase of the pulse (measured at 50% of height)
Width Ratio	Ratio between the systolic and diastolic widths (measured at 50% of height)
Variance	Deviation of signal from its mean value
Skewness	Degree of symmetry of a pulse
Kurtosis	Degree of sharpness of a pulse
Signal-to-Noise Ratio (SNR)	Level of PPG signal compared to noise
Zero-Crossing Rate (ZCR)	Number of times per second that the PPG signal crosses zero
Peak-To-Instantaneous Ratio (PIR)	Degree of change in amplitude over time

## Results

**Figure 3:**
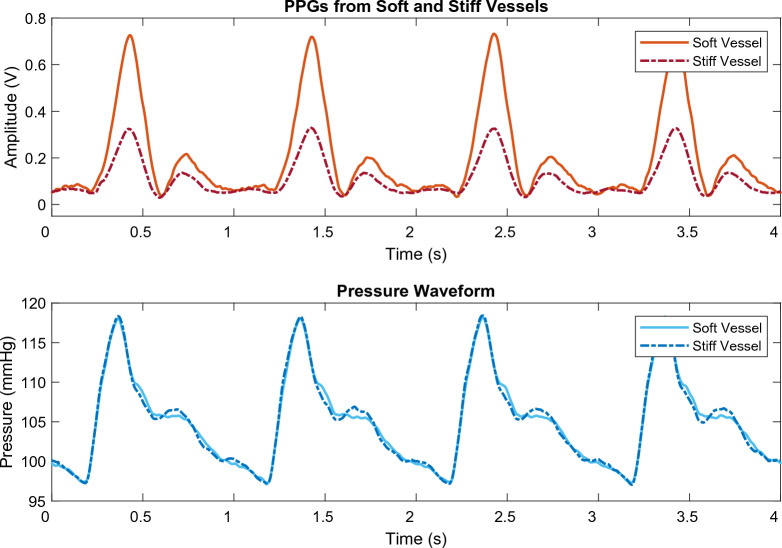
PPG and pressure signals from vessels with two different arterial stiffnesses. The PPG signals, which were obtained from sensors located on the vessel, depict the higher systolic and diastolic amplitudes of the soft vessel and the lower amplitudes of the stiff vessel. The pressure signals, conforming to a standard pressure pulse wave shape^41^, were obtained by sensors placed after the vessel and do not show much variation between stiffnesses. The soft vessel exhibited a Young Modulus of 0.6 MPa, while the stiff vessel had a Young Modulus 0.8 Mpa, representing the arterial stiffness values as highlighted in the literature^34–37^.

Red and infrared PPG signals were recorded from the custom vessels to observe differences in the signal due to varying arterial stiffness. Example red PPG signals from soft and stiff vessels are shown in Fig. 3, revealing morphological differences between them. There is a clear change in amplitude in both the systolic and diastolic peaks between the two vessels. The amplitude of the soft vessel systolic peak is 0.71 V and 0.21 V for the diastolic peak, while the stiff vessel systolic and diastolic peak amplitude was 0.32 V and 0.13 V, respectively.

**Figure 4:**
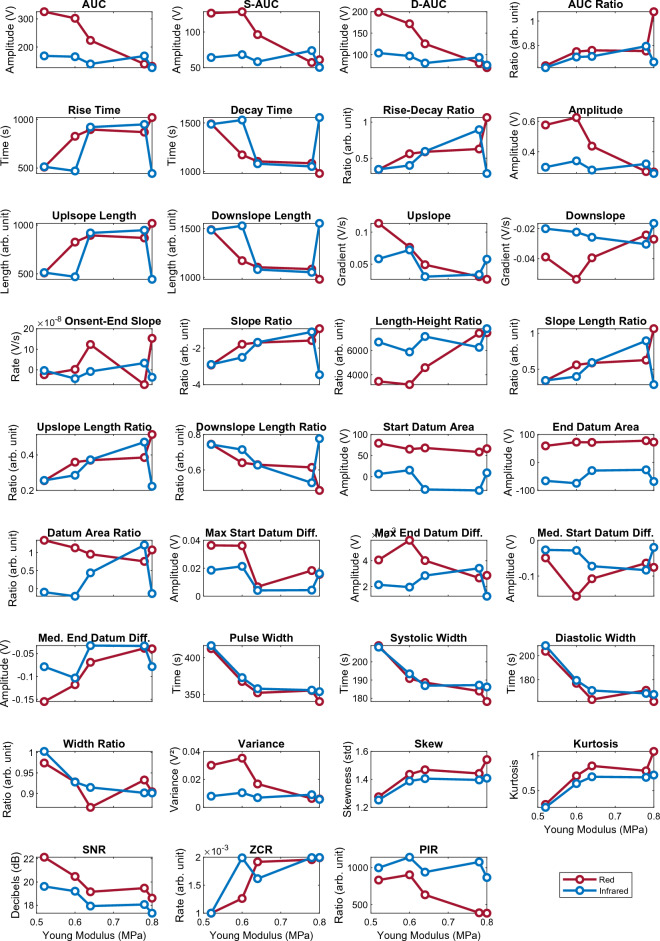
Line plots of the mean value of each feature, showing change in red and infrared PPG features with the custom vessels in order of increasing arterial stiffness.

The signals were analysed for further morphological differences, such as slope gradient and half peak width by feature extraction. The resultant features are displayed in simple line plots, using the mean value for each feature in Fig. 4, showing at a glance the trends in features across the five vessels, as stiffness increases. For example, in *Area Under Curve (AUC),* it can be seen that there is a strong negative correlation with vessel stiffness in the red PPG. Whereas a positive correlation is shown for both red and infrared signals in the *Skewness* and *Kurtosis* features. The features are also presented in box plot form in Fig. 4, depicting the range of values for each feature.

Pearson correlation coefficients were calculated to determine the degree of correlation in the features, as well as to identify any correlations which may not be visually striking in the plots. The resultant coefficients are ranked in Fig. 6 for the red and infrared PPG signal features, showing a different ranking order for the two wavelengths. Only correlation coefficients with a p-value below 0.05 are displayed, indicating statistical significance.

## Discussion

This paper has described the utilisation of custom vessels with varying stiffnesses to simulate vascular disease, followed by PPG signal acquisition and feature extraction on those vessels during pulsatile flow to look for significant markers in the PPG morphological features that may indicate stiffness. It has been our hypothesis that this can then be used for the detection of stiffening vessels that may indicate CVD. The vessels were created using an adjustable silicone elastomer and dip-coating process. Using this technique, vessels were created and placed in an in vitro test rig where the cardiovascular system could be emulated, consisting of a pulsatile pump and a surrounding vascular network circulating a blood-mimicking fluid, while acquiring PPG signals.

Visual observation of the PPG signals, exemplified in Fig. 3, indicates that the vessels and in vitro system in this study were successfully able to replicate human PPG signals, including the systolic and diastolic peaks, featuring the dicrotic notch. Furthermore, the morphology of these PPGs is evidently influenced when compared to a vessel with a higher arterial stiffness^42^. This change can be most profoundly seen in the reduction of the amplitude of the systolic peak in the stiffer vessel. A similar but lesser effect can also be noted in the diastolic peak amplitude. This initial observation can be explained by the physiology of blood flow and PPG acquisition. A stiffer artery is less able to expand; as such, a smaller change in blood volume is possible, resulting in a PPG with a lower amplitude^43^. This phenomenon can be linked to other aspects of the PPG wave—with a reduction in amplitude, a reduced a*rea under curve*, *pulse width* and *upslope/downslope* would be expected^44^. Cross-checking similar morphological features for expected trends as such provides a way of validating the feature extraction algorithm. For example, a decrease in *area under curve* is anticipated to result in a corresponding decrease in the *pulse width*.

Further analysis of the PPG signals was conducted by feature extraction to determine the magnitude of morphological changes with each vessel and reveal potential correlations between PPG features and arterial stiffness. The values of each extracted feature were compared across the five vessels of varying vessel stiffness in Fig. 4. A clear difference in PPG amplitudes with stiff and soft vessels is observed and it was expected that the *amplitude* feature, as well as *area under curve* and *pulse widths (systolic* and *diastolic)* would show a negative correlation, which is confirmed in the red line plots, helping to validate the feature extraction process. However, this correlation does not appear as strong in the *amplitude* and *area under curve* features of the infrared signal, as it does with *pulse width*, which is similar in both wavelengths. This suggests that the infrared signal is less susceptible to morphological changes, which may be linked to the absorption spectra of the methylene blue fluid, penetration depth of those wavelengths in this phantom or the placement of the PPG sensor on the vessel. *Rise time* was also expected to show a strong correlation with stiffness. Yet, although a statistically significant correlation is present, it does not rank highly in the feature ranking for red or infrared signals. Further experimentation is required to validate the statistical ranking of features. In the *width ratio*, a smoother negative trend is seen in the infrared signal. Overall, the red signal shows visibly larger changes than the infrared, particularly in *area under curve* (including *systolic* and *diastolic*) and *length-height ratio*. In other features, both wavelengths have a similar trend, as seen in *area under curve ratio, pulse width (including systolic and diastolic), skewness, kurtosis,* and *signal-to-noise ratio.* The varying nature of the extracted features between the two signals highlights the importance of multi-wavelength PPG monitoring, as it allows for simultaneous signal processing and physiological measurements which offer alternatives in situations where a particular wavelength may perform better or worse than the others.

**Figure 5:**
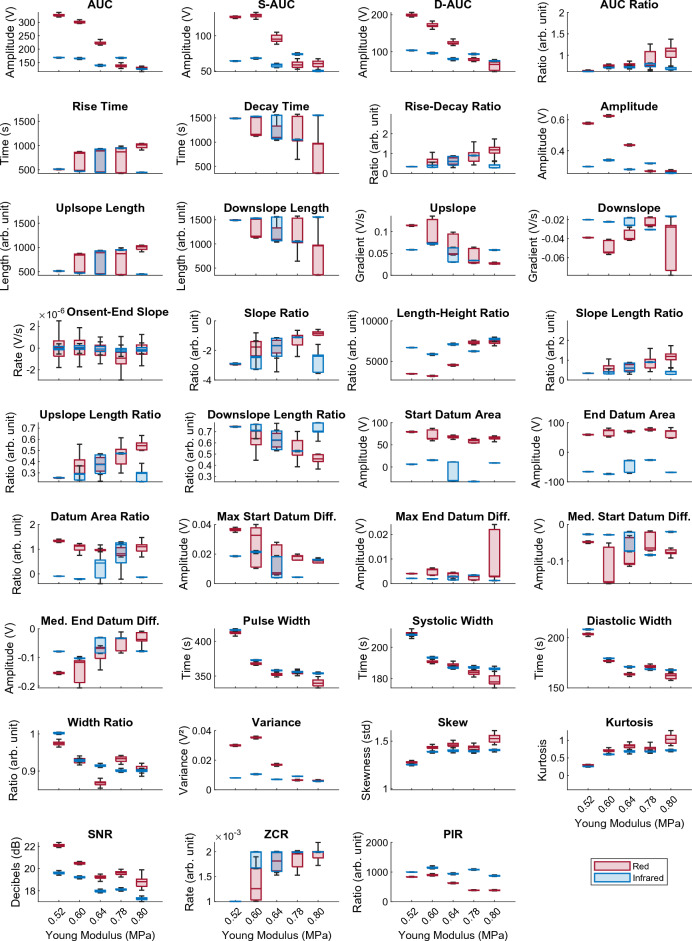
Box plots showing change in red and infrared PPG features with each of the custom vessels, in order of increasing arterial stiffness.

By observing the range and variability of the features, shown by the boxplots in Fig. 5, the reliable features can be determined. According to the boxplots, the most stable features are those related to area, amplitude, and width, as well as indexes such as *skewness, kurtosis, and signal-to-noise ratio* show notable change between stiffnesses while maintaining a low variation at each vessel. This suggests that these features can be used in a wide range of applications for non-invasive measurement, as low variability and high correlation would result in reliable arterial stiffness assessment, and possible other factors such as blood pressure. This hypothesis must be followed up with patient studies to compare pulse feature reliability in vivo. These results also showcase the value of signal indexes, for example pulse *skewness* and *kurtosis*, as they can be correlated with physiological phenomena such as vascular stiffening more strongly than some geometrical features^44,45^. These indexes can be used alongside geometrical features to better predict cardiovascular diseases.

**Figure 6:**
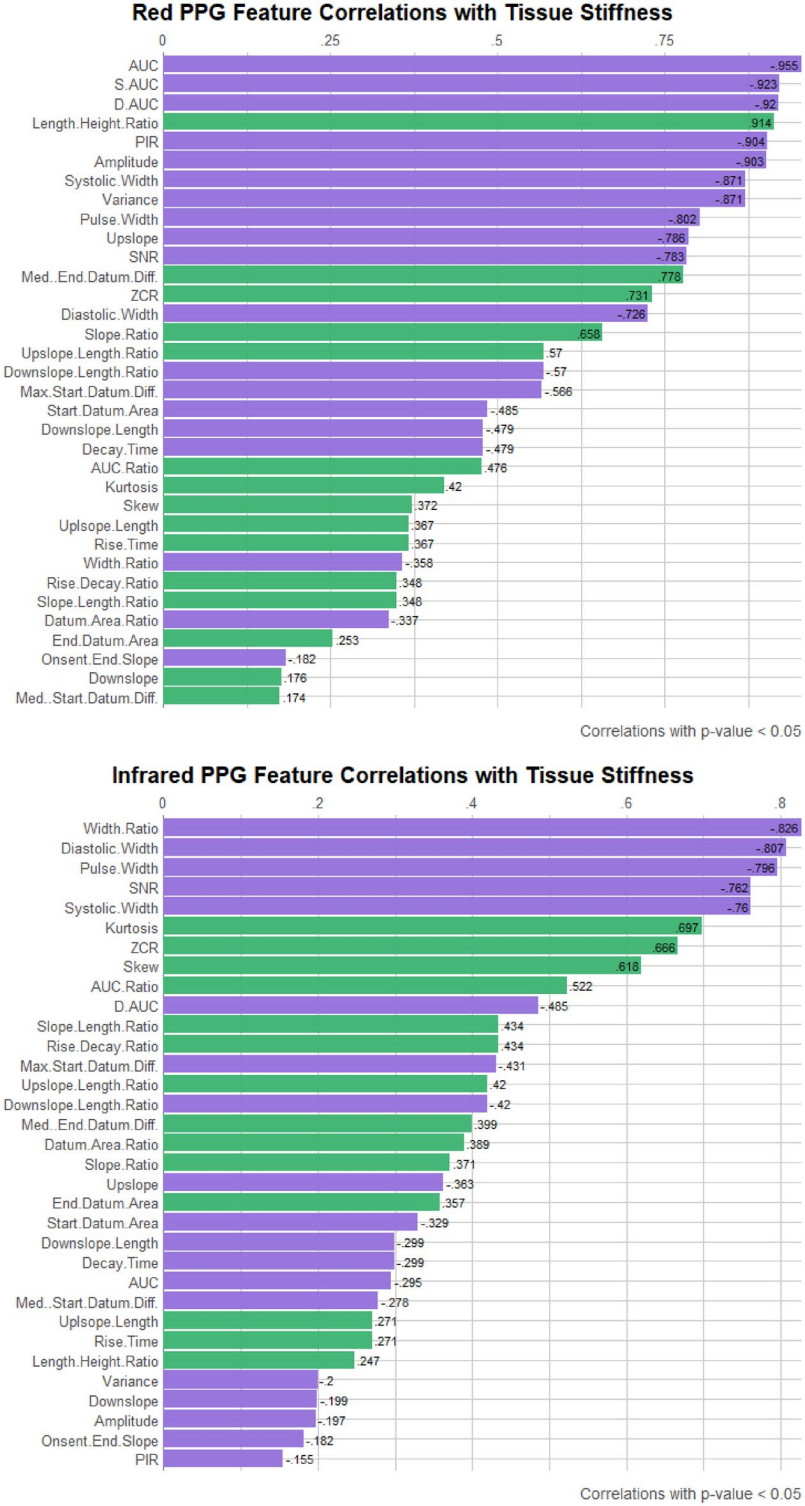
Red and infrared PPG features ranked by Pearson correlation coefficient with arterial stiffness. Only features which have a correlation coefficient with a corresponding p-value < 0.05 (statistically significant at 5% level) are displayed. (Green bars indicate positive correlation and purple bars indicate negative correlation).

A ranking based on correlation coefficients can signify the most reliable PPG features for arterial stiffness measurement, which can also be employed in in vivo and in vitro studies. As shown in Fig. 6, there is a significant link between arterial stiffness and the area of the PPG wave, as the three highest correlated features in red PPGs are *areas under the curve*. Interestingly, these features are not as highly ranked in infrared signals, further demonstrating the versatility of multi-wavelength measurement. The top three infrared PPG features are width related, suggesting that PPG wavelengths are affected differently by physiological changes such as arterial stiffness. To further discern the morphological differences, it is worth conducting investigations utilising other commonly used PPG wavelengths such as green light.

## Conclusion

This study has successfully demonstrated the use of PPG feature extraction and significance testing to identify morphological features affected by vessel stiffness in an in vitro environment. PPG signals were recorded from a range of vessels, resembling various stages of healthy and diseased arteries, which revealed distinct morphological changes correlated to arterial stiffness. Notably, red PPG signals showed the greatest shifts in area-related features, while pulse width infrared features were more strongly associated with arterial stiffness changes. This work expands the scope of non-invasive arterial stiffness evaluation and paves the way for further research involving more rigorous in vitro experiments, such as analysis at varying heart rates and flow rates to determine differences in responsiveness to flow changes between stiffnesses; as well as parallel in vivo studies to unravel the impact of biological factors such as age and respiration on PPG pulse shape.

## Data Availability

The datasets generated during and/or analysed during the current study are available from the corresponding author on reasonable request.
